# PSANet: Pyramid Splitting and Aggregation Network for 3D Object Detection in Point Cloud

**DOI:** 10.3390/s21010136

**Published:** 2020-12-28

**Authors:** Fangyu Li, Weizheng Jin, Cien Fan, Lian Zou, Qingsheng Chen, Xiaopeng Li, Hao Jiang, Yifeng Liu

**Affiliations:** 1 School of Electronic Information, Wuhan University, Wuhan 430072, China; 2019202120065@whu.edu.cn (F.L.); fce@whu.edu.cn (C.F.); zoulian@whu.edu.cn (L.Z.); 2019202120064@whu.edu.cn (Q.C.); xiaopengli2014@whu.edu.cn (X.L.); jh@whu.edu.cn (H.J.); 2 National Engineering Laboratory for Risk Perception and Prevention (NEL-RPP), Beijing 100041, China; liuyifeng3@cetc.com.cn

**Keywords:** 3D object detection, LiDAR, voxel, convolutional neural networks, autonomous driving

## Abstract

3D object detection in LiDAR point clouds has been extensively used in autonomous driving, intelligent robotics, and augmented reality. Although the one-stage 3D detector has satisfactory training and inference speed, there are still some performance problems due to insufficient utilization of bird’s eye view (BEV) information. In this paper, a new backbone network is proposed to complete the cross-layer fusion of multi-scale BEV feature maps, which makes full use of various information for detection. Specifically, our proposed backbone network can be divided into a coarse branch and a fine branch. In the coarse branch, we use the pyramidal feature hierarchy (PFH) to generate multi-scale BEV feature maps, which retain the advantages of different levels and serves as the input of the fine branch. In the fine branch, our proposed pyramid splitting and aggregation (PSA) module deeply integrates different levels of multi-scale feature maps, thereby improving the expressive ability of the final features. Extensive experiments on the challenging KITTI-3D benchmark show that our method has better performance in both 3D and BEV object detection compared with some previous state-of-the-art methods. Experimental results with average precision (AP) prove the effectiveness of our network.

## 1. Introduction

In recent years, convolutional neural networks (CNNs) have played a pivotal role in addressing the issues of object detection [1,2,3], semantic segmentation [4,5,6], and image super-resolution [7,8,9]. Although the average precision (AP) of 2D car detection is relatively considerable, autonomous driving is still a challenging task. As stated by Janai et al. [10], 3D object detection in the field of autonomous driving needs to find all objects in a given 3D scene, and determine their extent, direction, and classification. Therefore, the accuracy of 3D object detection directly impacts the safety and reliability of autonomous driving. As RGB images lack the necessary depth information, many researchers turn their attention to point cloud data, which retains accurate spatial information of objects. With the popularity of LiDAR and RGB-D cameras, the acquisition of point cloud data has become more convenient and feasible. However, point clouds are usually sparse, disordered, and unevenly distributed. How to effectively utilize the reliable information of the point cloud data for 3D object detection is a challenging task.

In the field of autonomous driving, data acquisition platforms are usually equipped with dual RGB color cameras and a LiDAR. The collected data includes the images taken by the left and right cameras and the point clouds scanned by LiDAR. Researchers can choose to use RGB images or point clouds for 3D object detection. Due to the modal difference between RGB image and point cloud data, many state-of-the-art 2D object detection methods cannot be directly applied to point clouds. For the RGB image and the corresponding point cloud data of a given scene, various strategies are proposed to solve the problem of 3D object detection. These schemes can be divided into the following three categories: (a) monocular image-based methods, which use RGB images containing rich color and texture information as the network input. However, in the process of converting a 3D scene into a 2D image by a color camera, the spatial depth information of the objects will inevitably be lost. Therefore, the performance of only using images for detection is far from reaching the safety standards for autonomous driving. (b) Multi-sensor fusion-based methods, most of which usually fuse point clouds with images through simple projections. As point clouds are usually sparse and unevenly distributed, it is difficult to ensure complete alignment when fusing with images. Although the point cloud data scanned by LiDAR contain accurate depth information, there are still few frameworks that can elegantly integrate multimodal data. (c) Point cloud-based methods, which use the original point clouds as input and extract the point-wise or voxel-wise features for detection. This kind of scheme shows excellent performance and even surpasses the methods based on multi-sensor fusion.

Recently, the voxel-based method has shown its unique speed advantage, and many advanced methods use it as their baseline. VoxelNet [11] is the pioneer of voxel-based methods. It proposes an end-to-end 3D object detection framework using point clouds as the only input. After dividing the point cloud space into regular voxels and extracting the voxel-wise features, a 3D backbone network is used to process these features for object detection. However, the computational cost of 3D CNN is too expensive to achieve the expected speed in the industrial field. For this reason, SECOND [12] proposes 3D sparse convolution for object detection and optimizes the 3D backbone network; this is a milestone that significantly improves the speed of network training and inference. Many follow-up works are carried out on this basis. For example, Pointpillars [13] and TANet [14] optimize the point cloud encoder and abandon the 3D backbone network, thereby further improving the inference speed of the network.

In this paper, we propose a novel detection framework called PSANet (Pyramid Splitting and Aggregation Network), which skillfully combines a 3D backbone network and a 2D backbone network. Inspired by TANet [14] and FPTNet [15], we propose a new 2D backbone network to complete the cross-layer fusion of multi-scale feature maps and extract robust features of BEV. Specifically, our proposed backbone network can be divided into a coarse branch and a fine branch. In the coarse branch, we use a pyramidal feature hierarchy to obtain multi-scale feature maps, which contain low-level features with rich texture information and high-level features with rich semantic information. This branch can effectively reduce the false detection caused by complex background and noise points. In the fine branch, we use our proposed pyramid splitting and aggregation (PSA) module to fuse different layers of multi-scale features cleverly. By fully fusing the feature maps of different levels, more expressive feature maps can be obtained, which enhances the robustness of the network. After merging these two branches, we can obtain a final feature map that integrates various information advantages for object detection. Experimental results on the KITTI dataset indicate that our detection framework has a good performance.

Benefiting from the rich information obtained by the deep fusion of multi-scale feature maps, our network can complete 3D and BEV detection tasks with high precision and achieve a good balance between the speed and the accuracy of detection. Specifically, our main contributions can be summarized as follows.

- We propose a new method to complete the cross-layer fusion of multi-scale feature maps, which uses the pyramid splitting and aggregation (PSA) module to integrate different levels of information.
- We propose a novel backbone network to extract the robust features from the bird’s eye view, which combines the advantages of cross-layer fusion features and original multi-scale features.
- Our proposed PSANet achieves competitive detection performance in both 3D and BEV detection tasks, and the inference speed can reach 11 FPS on a single GTX 1080Ti GPU.

## 2. Related Work

According to the representation of network input, 3D object detection methods can be divided into three categories: monocular image-based, multi-sensor fusion-based, and point cloud-based.

### 2.1. Monocular Image-Based Methods

Image-based 2D object detectors have been very mature, and the average precision can reach 94% on the KITTI-2D benchmark. As RGB images have the advantages of low cost, convenient acquisition, and easy processing, many researchers try to find some effective image-based 3D detection methods. Among them, the most concerned method is based on monocular images.

Mono3D [16] samples 3D candidate boxes in 3D space and projects them back to the image to generate 2D candidate boxes. These 2D candidate boxes are scored by using shape, context information, class semantics, instance semantics, and location. Then, a small number of high-quality object proposals are obtained by non-maximum suppression (NMS). As the exhaustive method is used to collect candidate boxes, a large number of proposals need to be searched in the 3D space, which causes certain efficiency problems.

GS3D [17] uses a 2D detector to predict the category, bounding box, and orientation of objects in RGB images. These detection results are used to guide the position and orientation of objects in 3D space. According to the prior knowledge of the scene, 3D guidance is generated by using 2D bounding boxes and projection matrix. After extracting the features of 3D guidance, the refined 3D bounding boxes can be obtained by using a 3D subnet. Compared with other 3D object detection methods based on monocular images, it can well balance the inference speed and detection accuracy. However, there is still a big gap between the detection performance and the safety standard of autonomous driving.

AM3D [18] combines the advantages of 3D reconstruction and proposes a novel monocular 3D object detection framework, which includes a 2D detector and a depth estimation network. It converts the 2D image into a 3D point cloud space to obtain pseudo-point clouds that are more conducive to detection, and then PointNet [19] performs 3D detection on the reconstructed pseudo-point clouds. To improve the recognition ability of point clouds, AM3D [18] proposes a multimodal feature fusion module, which complements the information of the RGB image with the pseudo-point cloud information. Unlike previous monocular image-based methods, it combines depth estimation information and significantly improves the detection performance.

### 2.2. Multi-Sensor Fusion-Based Methods

The point clouds of objects far away from LiDAR are sparse and difficult to distinguish, but these objects are very obvious in the image. Therefore, some methods based on multi-sensor fusion are proposed, and the most representative one is the fusion of point clouds and RGB images.

MV3D [20] takes the point clouds and RGB images as inputs. After projecting the point clouds to the bird’s eye view (BEV) and front view (FV), a 2D convolution neural network is used to extract image features and LiDAR multi-view features. As there are fewer occlusions in BEV, a small number of high-quality 3D proposals can be generated by using BEV features. Then, the multi-view features of the corresponding regions are deeply fused for object classification and detection. AVOD [21] further simplifies the input data, and only uses LiDAR BEV and RGB image for fusion. Moreover, a novel feature extractor is proposed to obtain high-resolution feature maps for small object detection. As point clouds are usually sparse, unevenly distributed, and may contain noise points, this fusion method cannot align the point clouds with the images well, which has a certain impact on the detection performance.

F-PointNet [22] improves the multi-sensor fusion method and proposes a 2D-detection-driven detector for 3D object detection. In the first stage, a 2D convolution neural network is used to generate 2D object region proposals in RGB images. In the second stage, these 2D region proposals are projected into the 3D point cloud space to form 3D viewing frustums. The point clouds in the 3D viewing frustums are divided into foreground objects and background objects. Moreover, only the segmented foreground points are used to predict objects. As this method relies too much on the performance of the 2D detector, it may lead to a wide range of missed detections.

ContFuse [23] proposes a novel fusion method for cameras and LiDAR, which realizes the precise positioning of 3D objects. This is an end-to-end trainable detection framework that uses a continuous fusion layer to encode discrete-state image features and continuous geometric structure information cleverly. As the multi-scale features of the image are fused into point cloud features, ContFuse [23] achieves competitive performance on the KITTI benchmark.

### 2.3. Point Cloud-Based Methods

Compared with RGB images, point cloud data with precise depth information can accurately estimate the 3D position of the object, which facilitates autonomous vehicles and robots to plan their behavior and paths. Due to the modal difference between point cloud data and RGB images, 2D CNNs cannot be directly used for point cloud processing. Therefore, PointNet-based [19,24] methods and voxel-based methods are proposed to process point clouds and complete 3D object detection.

The methods based on PointNet [19,24] usually extract point-wise features from the original point clouds and use a two-stage detector to classify the objects and predict the bounding boxes. In the first stage of PointRCNN [25], PointNet++ [24] extracts the features of the global point clouds and segments the foreground points belonging to the object. A small number of high-quality 3D proposals are generated by using the foreground points as the center. In the second stage, these 3D proposals are converted into a regular coordinate system and refined to obtain the final detection results. Although the performance of the PointNet-based [19,24] methods is very superior, it is difficult to guarantee the inference speed due to the huge amount of calculation for extracting the original point cloud features.

The voxel-based methods divide the 3D space into regular voxels or pillars and group the point clouds distributed in the space into corresponding voxels. After extracting the features of each voxel, the four-dimensional (4D) tensors representing the whole point cloud space are obtained by using sparse convolution middle layers, and an RPN [26] is used to implement the detection. VoxelNet [11] uses simplified PointNet [19] and voxel feature encoding (VFE) layers to extract voxel-wise features, and then a 3D convolution middle extractor is used to aggregate sparse four-dimensional tensors. To reduce the huge amount of calculation caused by 3D convolution, SECOND [12] applies sparse convolution to 3D object detection. As sparse convolution only operates on non-empty voxels, it dramatically improves the training and inference speed of the network.

Pointpillars [13] optimizes the encoder of SECOND [12] and encodes the point cloud space into pillars. Then, the simplified PointNet [19] is used to learn the features and convert the sparse 3D data into 2D pseudo-images for detection. Taking advantage of Pointpillars [13], TANet [14] studies the robustness of point cloud-based 3D object detection. A triple attention module is proposed to suppress the unstable point clouds, and the coarse-to-fine regression (CFR) module is used to refine the position of objects. After adding extra noise points, it can still ensure high-accuracy detection. However, due to the loss of point cloud information caused by spatial voxelization and insufficient utilization of 2D BEV information, the voxel-based method has a performance bottleneck, and the detection performance is not comparable to the PointNet-based [19,24] method.

## 3. PSANet Detector

In this section, we introduce the proposed PSANet detector, including the network architecture and implementation details.

### 3.1. Motivation

To solve the performance problem of the voxel-based method, we have investigated many related schemes and found that most of the current studies focus on how to reduce the information loss during spatial voxelization or design a two-stage detector to refine the results. Most of these detectors choose to simplify the RPN [26]. However, as an essential part of the 3D detector, an oversimplified RPN [26] will lose the details of the BEV information. For distant objects, they usually contain very sparse point clouds, and the detector is susceptible to the interference of noise points and background points, which may lead to false detections. Similarly, for objects that are severely truncated or occluded, the contour of their point clouds is usually incomplete. Therefore, it is necessary to determine the category according to the context information contained in the multi-scale feature maps.

PIXOR [27] proves that BEV information is beneficial for object detection in the field of autonomous driving. It converts point clouds into a BEV representation and designs a one-stage detector to complete high-precision detection of objects. Inspired by this, we propose a novel detector called PSANet and design a new backbone network to extract and fuse multi-scale BEV feature maps. The backbone network can be divided into two branches: a coarse branch and a fine branch. In the coarse branch, we extract features with different scales, including low-level features with rich texture information and high-level features with rich semantic information. In the fine branch, the PSA module implements the cross-layer fusion of multi-scale features and improves the expression ability of feature maps.

### 3.2. Network Architecture

**Figure 1:**
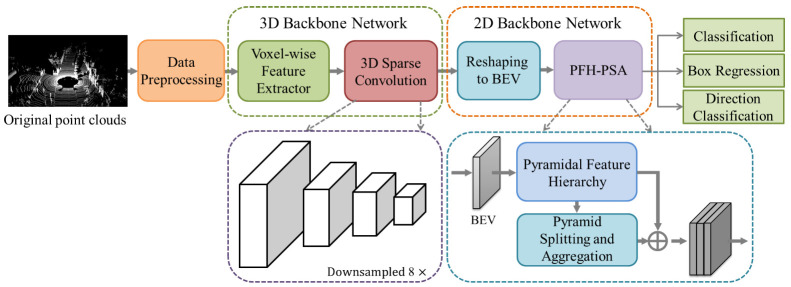
The structure of our PSANet. The detector divides the original point cloud space into regular voxels and extracts voxel-wise features by using a mean voxel-wise feature extractor. After the 3D sparse convolutional middle extractor learns the information along the *Z*-axis, the 3D sparse data is converted into a dense 2D bird’s eye view (BEV) pseudo-image. Finally, PFH-PSA completes the cross-layer fusion of multi-scale BEV features and obtains more expressive features for subsequent detection.

As shown in Figure 1, the proposed detector mainly includes five essential parts: (1) Data Preprocessing, (2) Voxel-wise Feature Extractor, (3) 3D Sparse Convolutional Middle Extractor, (4) Reshaping to BEV, and (5) Cross-Layer Fusion of Multi-Scale BEV Features (PFH-PSA).

### 3.3. Data Preprocessing

According to the coordinate transformation matrix, we project the point clouds into the image taken by the left camera and filter out the point clouds outside the image. As the original point clouds are usually irregularly distributed, the 3D convolutional neural network cannot directly process them. According to VoxelNet [11], we divide point cloud space into regular voxels. Specifically, for a given 3D scene, we only retain the part of point clouds that contain objects. The entire space is cropped to obtain an effective point cloud space within the range of D×H×W, where *D* represents the range of point clouds along the *Z*-axis (vertical direction), *H* represents the range of point clouds along the *Y*-axis (left and right of the car), and *W* represents the range of point clouds along the *X*-axis (the front of the car). For the KITTI dataset, we only process the point clouds within the range of [−3,1]×[−40,40]×[0,70.4] m${}^{3}$ and divide the cropped point clouds into voxels of size $v_{D}\times v_{H}\times v_{W}$. We choose 0.1×0.05×0.05 m${}^{3}$ as the voxel size, and a total of 40×1600×1408 voxels can be obtained. Generally, a high-definition point cloud scene may contain about 100k points. As the density of point clouds is related to many factors, the most common situation is that the point clouds of distant objects are usually very sparse. The number of points contained in different voxels varies greatly, so it is expensive to process all points in the voxel directly. For car detection, we set the number of points in each non-empty voxel not to exceed N(N=5). For voxels containing more than *N* points, we randomly sample *N* points to represent them. Conversely, for voxels that contain less than *N* points, we fill them with 0. This strategy brings two benefits: one is to use a small number of points to represent voxels, which greatly reduces the amount of computation, and the other is to avoid the negative impact caused by the unbalanced number of point clouds in different voxels.

### 3.4. Voxel-Wise Feature Extractor

We use a simple mean voxel-wise feature extractor to obtain the features of each voxel. Specifically, each non-empty voxel after data preprocessing contains *N* points, and we average the information of these *N* representative point clouds and take the results as the voxel-wise features. Remarkably, although the mean voxel-wise feature extractor has a simple structure, it can effectively extract features and avoid using complex PointNet [19] to extract voxel-wise features.

### 3.5. 3D Sparse Convolutional Middle Extractor

**Figure 2:**
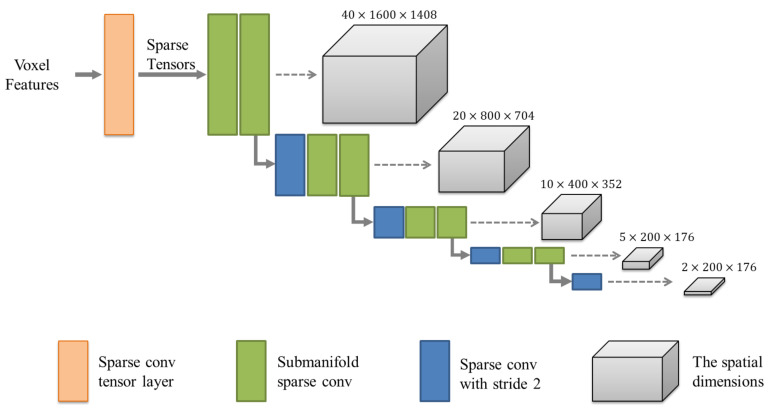
The structure of the 3D sparse convolutional middle extractor. The orange box is the sparse convolutional tensor layer, which is used to convert voxel features into 4D sparse tensors. The blue boxes are sparse convolutional layers with stride =2, and the green boxes are submanifold convolutional layers. The gray cuboids are used to show the changes in the spatial dimension.

To improve the computational efficiency of 3D convolution, we use the sparse convolution proposed by SECOND [12] to process non-empty voxels. As shown in Figure 2, we take the voxel-wise features obtained by mean voxel-wise feature extractor as input and then convert them into four-dimensional (4D) sparse tensors by using a sparse convolutional tensor layer. The 4D sparse tensors can be expressed as $C^{\prime }\times D^{\prime }\times H^{\prime }\times W^{\prime }$, where $C^{\prime }$ is the number of channels, and the initial $D^{\prime }$, $H^{\prime }$, and $W^{\prime }$ are $\frac{D}{v_{D}}$, $\frac{H}{v_{H}}$, and $\frac{W}{v_{W}}$, respectively. Then, the sparse tensor representing the whole space is eight times downsampled by sparse convolutional layers and submanifold convolutional layers. In this process, the network learns the information along the *Z*-axis in space and downsamples the *Z*-dimensionality to 2. This part implements the compression of height, which facilitates converting sparse 3D data into dense 2D pseudo-images.

### 3.6. Reshaping To BEV

We use the 4D tensor expressed as $C^{\prime }\times D^{\prime }\times H^{\prime }\times W^{\prime }$ to represent the sparse data that is downsampled eight times, where $C^{\prime }$ is the number of channels, and $D^{\prime }\times H^{\prime }\times W^{\prime }$ is the spatial dimension of sparse data. After the dense operation on the 4D sparse tensor, the dimensions of $C^{\prime }$ and $D^{\prime }$ are fused to obtain a 2D pseudo-image for PFH-PSA. The point cloud space with a shape of 128×2×200×176 is mapped to the BEV pseudo-image with a shape of 256×200×176.

### 3.7. Cross-Layer Fusion of Multi-Scale BEV Features (PFH-PSA)

**Figure 3:**
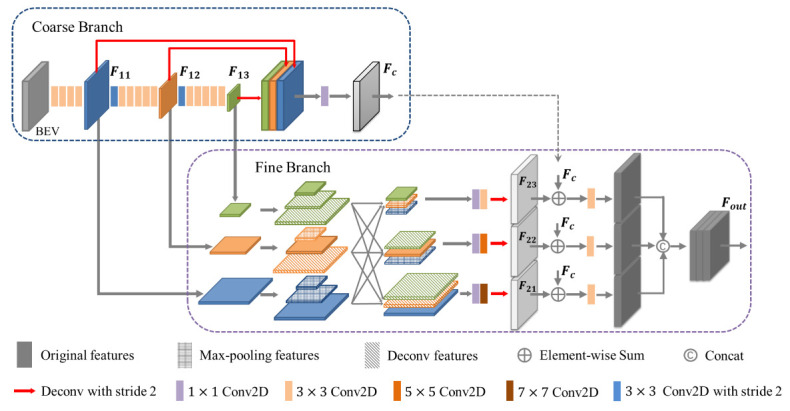
The structure of our proposed backbone network PFH-PSA. The red arrows indicate deconvolution layers, purple boxes indicate 1×1 convolutional layers, light yellow boxes indicate 3×3 convolutional layers, orange boxes indicate 5×5 convolutional layers, brown boxes indicate 7×7 convolutional layers, and blue boxes indicate the convolutional layers with stride = 2. Different colors indicate feature maps with different scales, and different textures indicate feature maps obtained through deconvolution layers or max-pooling layers.

RPN [26] is an important part of many high-precision object detectors, which directly affects the detection performance. To this end, we propose a novel backbone network to implement the cross-layer fusion of multi-scale BEV features, so as to make full use of the advantages of various features. As shown in Figure 3, the backbone network contains two branches: a coarse branch and a fine branch. The coarse branch is composed of a pyramidal feature hierarchy (PFH), and the fine branch is composed of a pyramid splitting and aggregation (PSA) module. In this paper, the backbone network can be referred to simply as PFH-PSA. Specifically, in the coarse branch, several consecutive convolutional layers with strides of 1 and 2 are used to obtain multi-scale feature maps. We can obtain the feature maps $F_{11}$, $F_{12}$, and $F_{13}$ with sizes *S*, S/2, and S/4, respectively. Then, the multi-scale feature maps are deconvolved back to the same size *S* and fused to obtain the output $F_{c}$ containing multiple information. In the fine branch, the multi-scale feature maps of the coarse branch are sampled to reconstruct three new pyramidal feature hierarchies. This whole process is implemented by deconvolution layers and max-pooling layers, and the feature maps with the corresponding size are fused to form a reorganized pyramidal feature hierarchy. Finally, the multi-scale feature maps are deconvolved to $F_{21}$, $F_{22}$, $F_{23}$ with the same size, and fused with $F_{c}$ as the final feature $F_{out}$.

The pyramidal feature hierarchy contains low-level features with rich texture information and high-level features with rich semantic information, which can effectively avoid false detections caused by background points and noise points. Moreover, the multi-scale feature maps have different resolutions and receptive fields, which is conducive to the detection of small objects. The pyramid splitting and aggregation module implements the cross-layer fusion of multi-scale feature maps and obtains more expressive feature maps. After element-wise summing the outputs of these two branches, we can obtain a feature map that combines various information for the detection task.

The following are the implementation details. We use a BatchNorm layer and a ReLU layer after each convolutional layer, so we can use Conv2d($C_{in}$, $C_{out}$, K, S) to represent the Conv2D-BatchNorm-ReLU layer, where $C_{in}$ is the number of input channels, $C_{out}$ is the number of output channels, K is the size of the convolutional kernel, and S is the stride. We take the BEV pseudo-image with a shape of $\left(C^{\prime }\times D^{\prime }\right)\times H^{\prime }\times W^{\prime }$ as input, the number of channels is $(C^{\prime }\times D^{\prime })=256$, and the scale is $H^{\prime }\times W^{\prime }=200\times 176$. In the coarse branch, the BEV pseudo-image generates multi-scale feature maps through three blocks. The first block contains a Conv2d(256, 128, 3, 1), which reduces the number of channels to 128, and then three continuous Conv2d(128, 128, 3, 1) are used to obtain the feature map $F_{11}$. The second block contains a Conv2d(128, 256, 3, 2) for downsampling $F_{11}$, and then we use five continuous Conv2d(256, 256, 3, 1) to obtain $F_{12}$. The third block is identical to the second block, and the feature map $F_{13}$ with a size of S/4 is obtained. For $F_{11}$, $F_{12}$, and $F_{13}$, we also use three blocks to implement upsampling. These three blocks are composed of deconvolution layers with stride 1, 2, and 4. Each deconvolution layer is followed by a BatchNorm layer and a ReLU layer. We use a Conv2d (768, 256, 1, 1) to reduce the number of channels and obtain the output $F_{c}$ of the upper half branch. The coarse branch has the following forms,

(1) $$F_{c}=W_{1\times 1}⊗\left(U^{1}\left(F_{11}\right)⊕U^{2}\left(F_{12}\right)⊕U^{4}\left(F_{13}\right)\right)$$

where $W_{1\times 1}⊗$ represents the 1×1 convolutional layer, $U^{\beta }$ represents the deconvolution layer with stride =β, and ⊕ represents concatenation.

In the fine branch, we use deconvolution layers and max-pooling layers to process $F_{11}$, $F_{12}$, and $F_{13}$, and generate feature maps with three different scales. Then, we concatenate the feature maps of the corresponding size to form a new pyramidal feature hierarchy, and the number of channels is 256, 512, and 640, respectively. We use three 1×1 convolutional layers to reduce the number of channels, and then we use kernels of sizes 3×3, 5×5, and 7×7 to process these multi-scale feature maps and obtain different receptive fields. To ensure the calculation speed, we use two 3×3 convolutional layers instead of the 5×5 convolutional layer and three 3×3 convolutional layers instead of the 7×7 convolutional layer. After deconvolution layers with stride 1, 2, and 4, we can get $F_{21}$, $F_{22}$, and $F_{23}$ with the same size. We sum them with $F_{c}$ to complete the fusion of the two branches. Then, a Conv2d(256, 256, 3, 1) is used to further fuse the features. Finally, we concatenate the above features to get the final output $F_{out}$. This process is called the pyramid splitting and aggregation module, which has the following form:

(2) $$F_{21}=W_{7\times 7}⊗\left(W_{1\times 1}⊗\left(F_{11}⊕U^{2}\left(F_{12}\right)⊕U^{4}\left(F_{13}\right)\right)\right)$$

(3) $$F_{22}=W_{5\times 5}⊗\left(W_{1\times 1}⊗\left(D^{2}\left(F_{11}\right)⊕F_{12}⊕U^{2}\left(F_{13}\right)\right)\right)$$

(4) $$F_{23}=W_{3\times 3}⊗\left(W_{1\times 1}⊗\left(D^{4}\left(F_{11}\right)⊕D^{2}\left(F_{12}\right)⊕F_{13}\right)\right)$$

(5) $$F_{out}=\left(W_{3\times 3}⊗\left(F_{21}+F_{c}\right)\right)⊕\left(W_{3\times 3}⊗\left(F_{22}+F_{c}\right)\right)⊕\left(W_{3\times 3}⊗\left(F_{23}+F_{c}\right)\right)$$

where $W_{\alpha \times \alpha }⊗$ represents the α×α convolutional layer, $U^{\beta }$ represents the deconvolution layer with stride =β, $D^{\gamma }$ represents the max-pooling layer with kernel size =γ, ⊕ represents concatenation, and + represents element-wise summation.

In the detection head, we inherit the method proposed by SECOND [12] and determine the fixed-size anchors according to the average size of the ground truths in the KITTI dataset. We choose a set of anchors with a size of $w\times l\times h=1.6\times 3.9\times 1.56m^{3}$ to detect cars. Finally, we use three 1×1 convolutional layers to implement object classification, bounding box regression, and direction classification.

### 3.8. Loss Function

Our loss function $L_{total}$ consists of three parts: Focal loss $L_{cls}$ for object classification, Smooth-L1 loss $L_{reg}$ for angle and position regression, and Softmax loss $L_{dir}$ for direction classification.

In one-stage detectors, the proportion of positive and negative samples is extremely unbalanced. To reduce the weight of negative samples during training, RetinaNet [28] proposes effective Focal loss. We use it as our classification loss, which has the following form,

(6) $$L_{cls}=-\alpha_{t}{\left(1-P_{t}\right)}^{\gamma }log\left(P_{t}\right)$$

where $P_{t}$ is the model’s estimated probability for the corresponding bounding box, and α and γ are the hyperparameters of the loss function. We use α=0.25 and γ=2.

Regression loss includes angle regression and bounding box regression. For the anchor used for detection, its center can be expressed as $x_{a}$, $y_{a}$, $z_{a}$, and its length, width, and height can be expressed as $l_{a}$, $w_{a}$, and $h_{a}$, respectively. In addition, we define the yaw rotation around the *z*-axis as $\theta_{a}$. Therefore, the bounding box can be expressed as $\left[x_{a},y_{a},z_{a},l_{a},w_{a},h_{a},\theta_{a}\right]$. Correspondingly, the bounding box of the ground truth can be expressed as $\left[x_{g},y_{g},z_{g},l_{g},w_{g},h_{g},\theta_{g}\right]$. The subscripts *a* and *g* are used to distinguish between the anchor and the ground truth, respectively. We define the seven regression targets $\left[\Delta_{x},\Delta_{y},\Delta_{z},\Delta_{l},\Delta_{w},\Delta_{h},\Delta_{\theta }\right]$ as follows,

(7) $$\Delta x=\frac{x_{g}-x_{a}}{\sqrt{{\left(l_{a}\right)}^{2}+{\left(w_{a}\right)}^{2}}},\Delta y=\frac{y_{g}-y_{a}}{\sqrt{{\left(l_{a}\right)}^{2}+{\left(w_{a}\right)}^{2}}},\Delta z=\frac{z_{g}-z_{a}}{h_{a}},$$

(8) $$\Delta l=\log\left(\frac{l_{g}}{l_{a}}\right),\Delta w=\log\left(\frac{w_{g}}{w_{a}}\right),\Delta h=\log\left(\frac{h_{g}}{h_{a}}\right),$$

(9) $$\Delta \theta =\sin(\theta_{g}-\theta_{a}),$$

The regression loss has the following form,

(10) $$L_{reg}=\sum_{u\in [x,y,z,l,w,h,\theta ]}SmoothL1\left(\Delta u\right)$$

The total loss function for training is as follows,

(11) $$L_{total}=\beta_{1}L_{cls}+\beta_{2}L_{reg}+\beta_{3}L_{dir}$$

where $L_{dir}$ is the Softmax loss for direction classification. $\beta_{1}$, $\beta_{2}$, and $\beta_{3}$ are hyperparameters, and we use $\beta_{1}=1.0$, $\beta_{2}=2.0$, and $\beta_{3}=0.2$.

## 4. Experiments

In this section, our PSANet is trained and evaluated on the challenging KITTI-3D benchmark [29]. First, we compare the performance of 3D and BEV object detection with other methods and then we list some ablation experiments to prove the effectiveness of our network. Finally, we show some visualizations of detection results and compare them with some state-of-the-art voxel-based methods.

### 4.1. Dataset

In the field of autonomous driving, the KITTI dataset is currently the world’s largest dataset for evaluating 3D object detection algorithm. Specifically, it contains real image data collected from different scenes such as urban areas, rural areas, and highways. As each image may contain up to fifteen cars, object detection on the KITTI dataset becomes a very challenging task. We train and evaluate our network using the KITTI dataset, which contains 7481 pairs of training samples and 7518 pairs of test samples. As the ground truth of the test set is not public, we use the method proposed by MV3D [20] to divide the 7481 pairs of samples into a training set containing 3712 samples and a validation set containing 3769 samples. According to the occlusion level, the degree of truncation, and the height of the bounding box in the 2D image, the KITTI benchmark divides objects into three difficulty levels: easy, moderate, and hard. Therefore, we evaluate the performance of the detector from these three different levels of difficulty. All the following experiments are performed on a single GTX 1080 Ti GPU.

### 4.2. Implementation Details

#### 4.2.1. Network Details

For car detection, we select the point cloud space in the range of D×H×W=[−3,1]×[−40,40]×[0,70.4] m${}^{3}$ as the network input. Moreover, we use $v_{D}\times v_{H}\times v_{W}=0.1\times 0.05\times 0.05$ m${}^{3}$ as the voxel size and divide the cropped point cloud space into 40×1600×1408 voxels. To ensure the computational efficiency of training and inference, we set each voxel to contain no more than N(N=5) points. During training, we stipulate that the entire space contains no more than 16,000 non-empty voxels. For scenes where the number of voxels exceeds the specified maximum number, we use random sampling to process them. Similarly, during the test, we stipulate that the scene contains no more than 40,000 non-empty voxels. Finally, we choose w×l×h=1.6×3.9×1.56 m${}^{3}$ as the anchor size.

#### 4.2.2. Training Details

**Figure 4:**
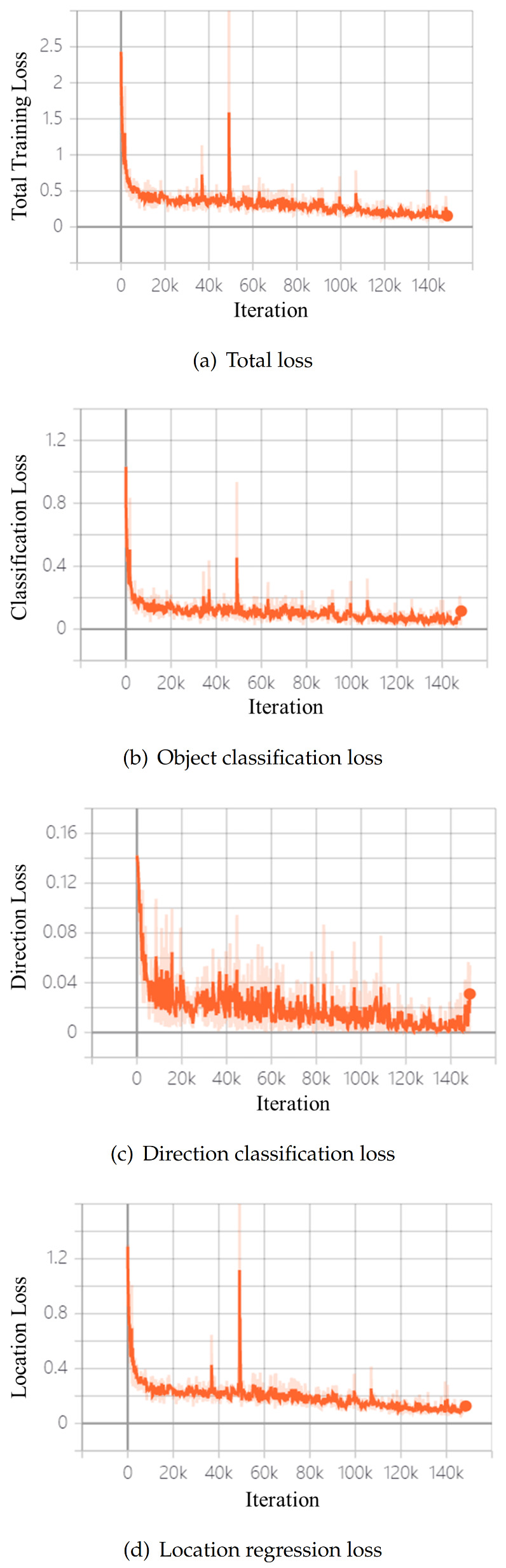
The changes of loss value during network training.

During the training process, we use Kaiming initialization to configure the parameters of our network. The initial learning rate is 0.0003, and we use an Adam optimizer to train the network on a single GTX 1080Ti GPU with a batch size of 2. Our proposed network is trained for 80 epochs (150k iterations), and it takes 27 h in total. We use the value of beta1 is 0.9, the value of beta2 is 0.999, and the value of epsilon is 10 × 10${}^{-8}$. The total loss during the entire training process is shown in Figure 4, where the abscissa is the number of iterations, and the ordinate is the loss value. It can be seen from Figure 4a that the total loss of the network converges relatively well. Figure 4b–d show the object classification loss, direction classification loss, and location regression loss, respectively.

**Figure 5:**
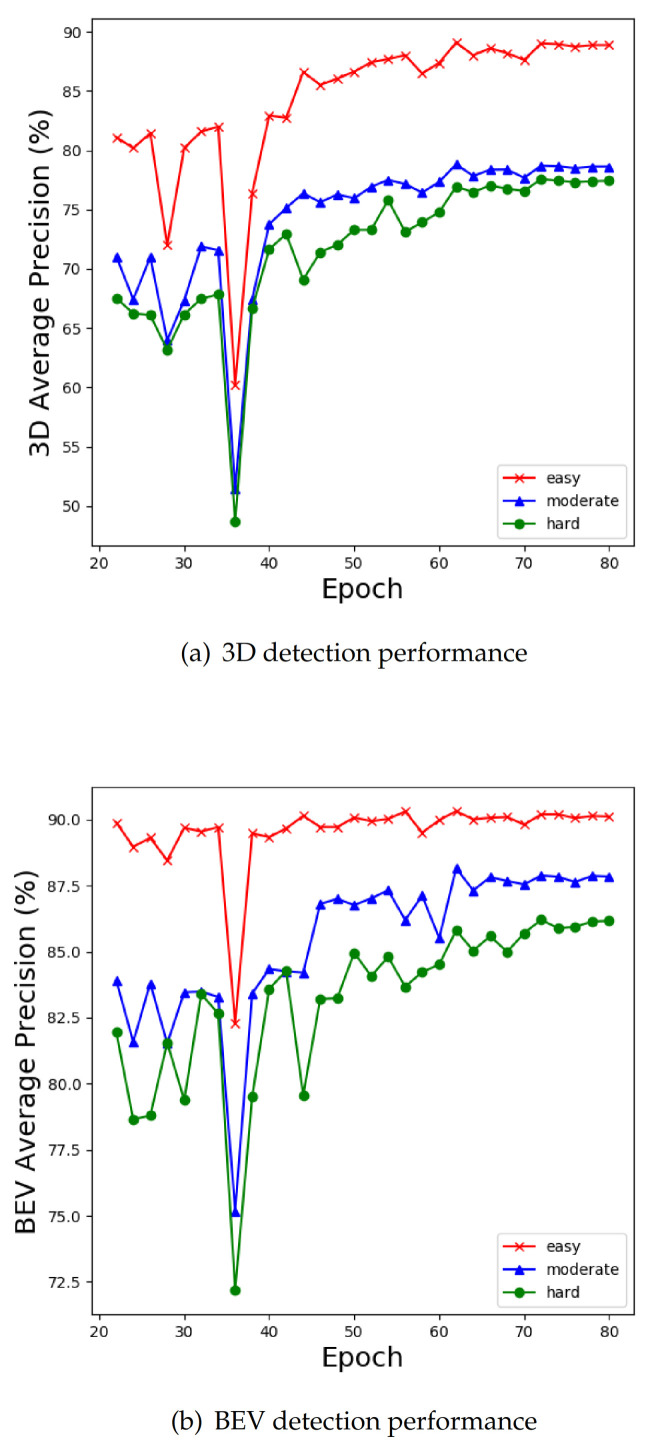
The 3D and BEV average precision (AP) (%) of different epochs on the KITTI validation set.

As the test set is not public, we divided 7481 pairs of labeled samples to obtain a training set containing 3712 samples. To prevent the network from overfitting due to too few training samples, we augment the database according to SECOND [12], including random flip, global rotation, and global scaling. Moreover, we sample ground truths from the training set to build an augmented database that includes labels and point clouds in ground truths. During training, several ground truths are randomly selected from the augmented database and spliced into the real point cloud scene being trained. Here, we constrain the spliced ground truth and the real ground truth to have no intersection. To show the variation tendency of the detector performance more intuitively, we save the model parameters in different epochs during the training process and draw two performance line graphs. As shown in Figure 5, our network has no obvious overfitting, and the final performance remains stable at a high level.

### 4.3. Comparisons on the KITTI Validation Set

We compare our PSANet with some previous state-of-the-art methods, which are the most representative algorithms in recent years. According to the structure, these methods can be divided into two categories, one is based on the two-stage detector, and the other is based on the one-stage detector. According to the representation of the input data, some of them are based on point cloud and image fusion, while others only use point clouds as input. It is worth noting that our PSANet is a one-stage detector that only takes point clouds as input. The detailed comparison is shown in Table 1. We conduct a comprehensive comparison from the two aspects of 3D and BEV object detection, in which the boldface indicates the best performance among the current evaluation indicators.

KITTI uses the 3D object detection average precision of moderate difficulty as the most important evaluation criterion, but the detection of hard objects is more challenging. From Table 1, we can find that our PSANet achieves the best performance in different levels of 3D object detection tasks, and even surpassing the two-stage methods. In the task of BEV object detection, our method is very close to the current optimal method and obtains sub-optimal results.

**Figure 6:**
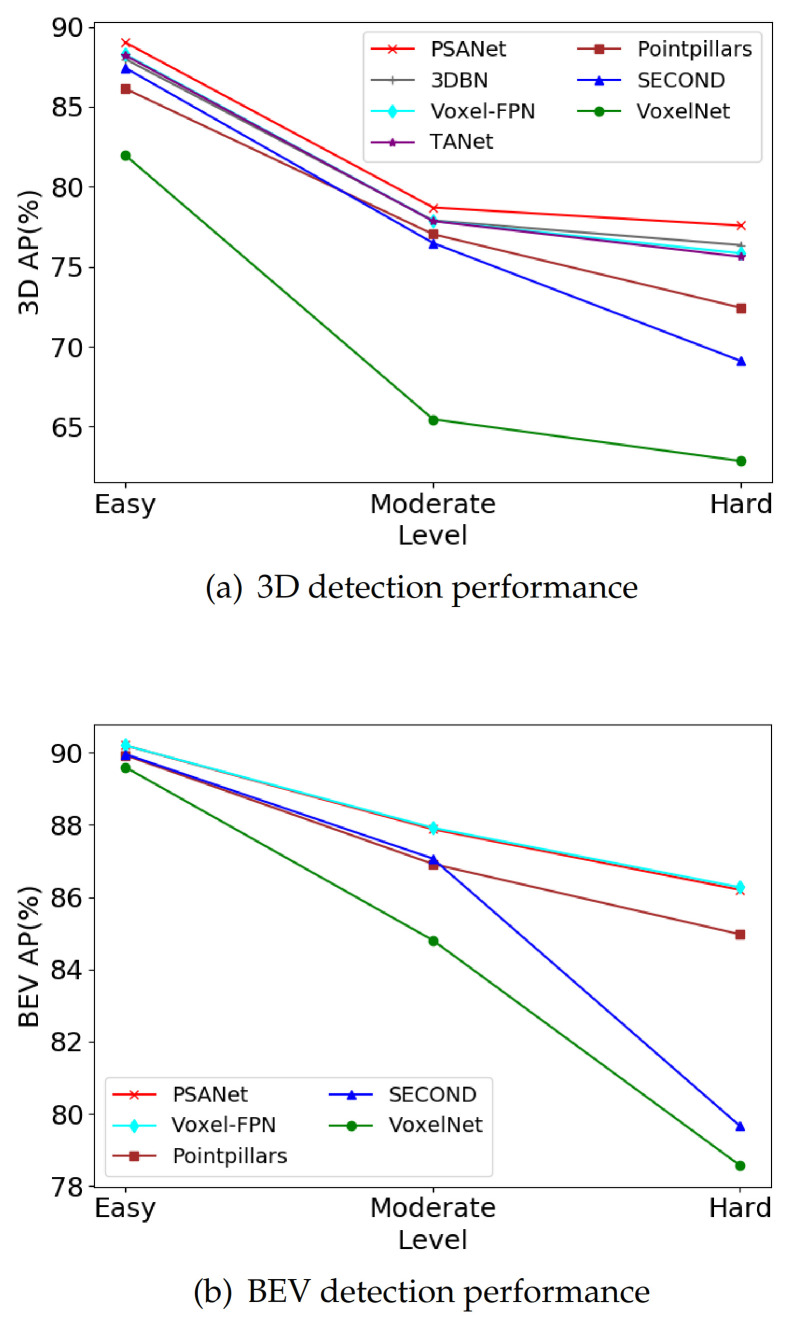
The average precision (AP) (%) of different methods on the KITTI validation set.

To show the superiority of our method more intuitively, we draw the performance line charts of 3D and BEV detection tasks. As shown in Figure 6, the abscissa represents different levels of difficulty, and the ordinate represents the average precision of 3D and BEV object detection. As can be seen from Figure 6a, our method is significantly better than other one-stage algorithms in 3D object detection tasks. Moreover, by comparing the slope of the broken line between the moderate level and hard level, we can find that when the detection difficulty increases, the performance of our method will not decrease significantly, which further indicates that our network is more robust. Furthermore, as shown in Figure 6b, our method also achieves outstanding performance in the BEV object detection task, which is almost comparable to the most advanced methods.

### 4.4. Ablation Studies

#### 4.4.1. Different Backbone Networks

Our proposed backbone network integrates multi-scale and cross-layer features. To prove its superiority, we test the influence of different backbone networks on detection performance. We use SECOND [12] as our baseline, which uses the most common structure as the backbone network. It downsamples the BEV features once, then converts the two feature maps to the same size through deconvolution layers, and the result is directly used for detection. Our coarse branch further downsamples the BEV features, and fuse the multi-scale feature maps for detection. After generating multi-scale feature maps, the fine branch completes the cross-layer fusion of the obtained features, but the original multi-scale features will not be fused. Our network uses a new structure that combines these two branches. This structure not only uses the fine branch to fuse the multi-scale feature maps but also retains the independent fusion of the coarse branch. The detailed experimental results are shown in Table 2, where PFH indicates the coarse branch, and PSA indicates the fine branch. We bold the relevant values to highlight the optimal value of each metric.

It can be seen from the above table that only adding the coarse branch to obtain multi-scale feature maps can hardly improve the performance of the detector, which also explains why the latest detectors choose to simplify the structure of multi-scale feature extraction. When the fine branch is added to the backbone network, the PSA module deeply integrates the texture information of the low-level features and the semantic information of the high-level features. It greatly enhances the expressive ability of feature maps, thereby significantly improving the performance of BEV detection. However, after discarding the coarse branch, the average precision of the 3D object detection is slightly reduced, which indicates that the coarse branch output $F_{c}$ contains the information required for 3D detection. When the two branches work at the same time, the accuracy of 3D and BEV object detection is significantly improved, which proves that these two branches have their advantages for different detection tasks, and their advantages have a synergistic effect.

#### 4.4.2. Different Fusion Methods

**Figure 7:**
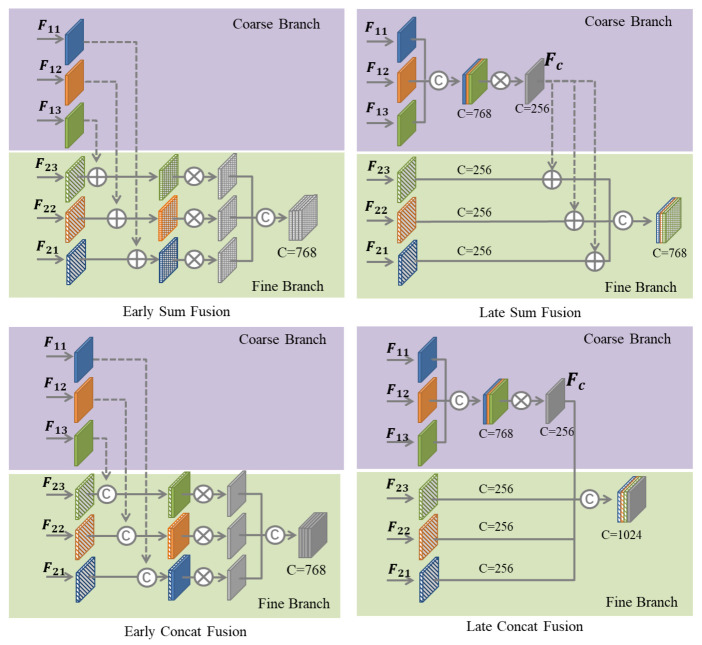
Different fusion methods of the two branches, where C represents concatenation, ⊕ represents the element-wise summation, and ⊗ represents the convolutional layer.

The feature maps obtained from the two branches contain effective information for 3D and BEV object detection. To effectively fuse them and obtain features with stronger expressive ability, we design four different fusion methods to fuse the advantages of these two branches. At present, the most common fusion method is the element-wise summation and channel concatenation. As the two branches contain six outputs, direct concatenation will cause a certain computational burden. We fuse the coarse branch separately to obtain $F_{c}$ and then fuse it with each output of the fine branch. Therefore, according to whether the coarse branches are fused separately, we divide these four fusion methods into early fusion and late fusion. As shown in Figure 7, the method using $F_{c}$ is called late fusion, and the opposite is called early fusion. Table 3 shows the impact of different fusion methods on performance. We bold the relevant values to highlight the optimal value of each metric.

From Table 3, we can find that in the same case of early fusion or late fusion, the channel concatenation fusion method is more conducive to BEV detection, while the element-wise summation is more conducive to 3D object detection. Similarly, when using concatenation or element-wise summation, we can see that the later fusion method is always better than early fusion. Compared with BEV detection, this article pays more attention to the performance of 3D object detection, so we choose the late sum fusion method as the final model of the network.

### 4.5. Analysis of the Detection Results

#### 4.5.1. Detection Results on the KITTI Validation Set

**Figure 8:**
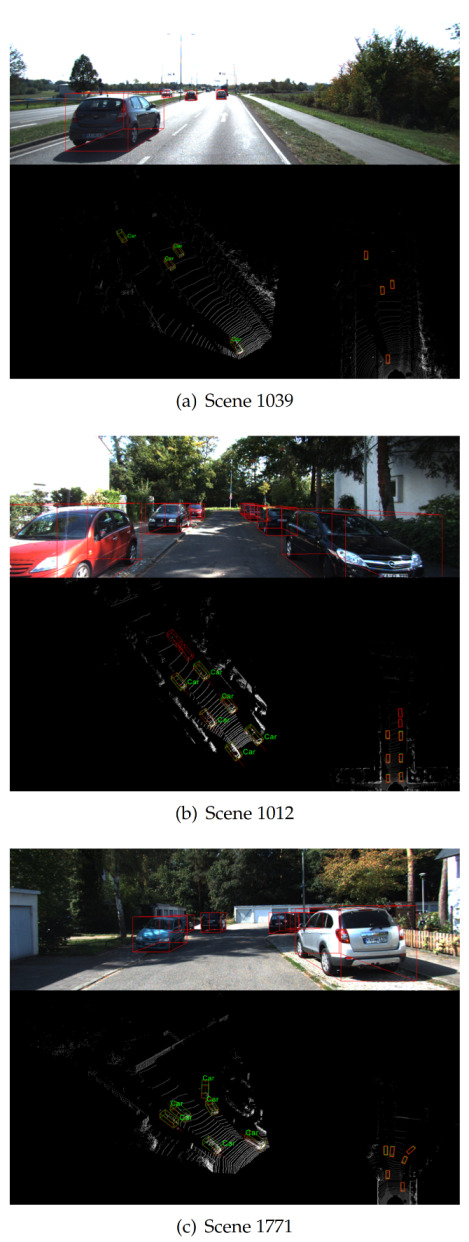

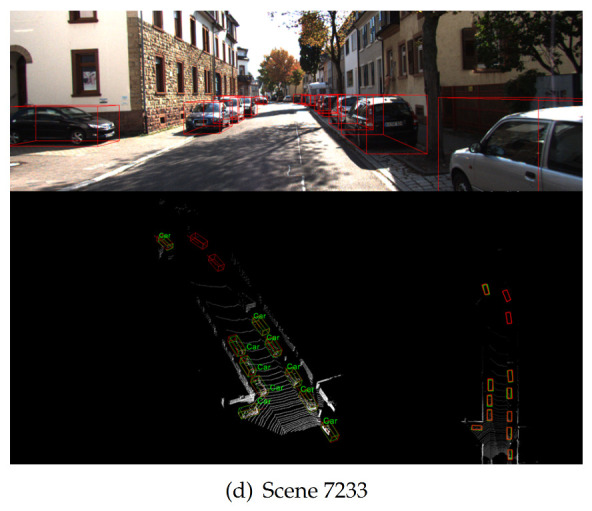
Detection results on the KITTI validation set, where the green boxes represent the bounding boxes of the ground truth, the red boxes represent our detection results, and the short red lines indicate the results of our direction classiﬁcation.

We draw the bounding box of the ground truth and our detection results with green lines and red lines, respectively. Each detection bounding box has a short red line to indicate the result of our direction classification. To observe the results more intuitively, we project the detection results from the point cloud space to the RGB image and generate the corresponding bounding boxes. Each scene in Figure 8 contains three parts: the top shows the 3D bounding boxes in the RGB image, the picture on the bottom left is the visualization of the detection results in real point cloud space, and the picture on the bottom right is the results of BEV detection.

As shown in Figure 8a, the cars in this scene are not severely occluded or truncated, and these targets can be easily detected. Figure 8b,c have several heavily occluded cars. Although they are ignored by the ground truth, we can still accurately detect them and determine their direction. Figure 8d shows the detection results of a complex scene. The cars in this scene are arranged very densely, and there are several occluded and partially truncated cars. Nevertheless, we can still complete accurate detection.

#### 4.5.2. Comparison with Some State-of-the-Art Voxel-Based Methods

**Figure 9:**
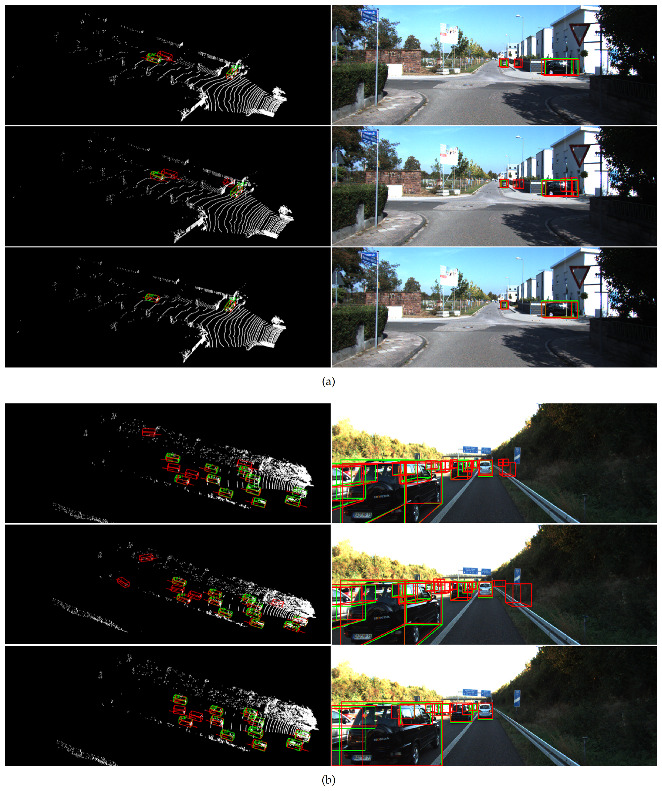
Comparison of detection results from Pointpillars [13] (**top**), SECOND [12] (**middle**), and ours (**bottom**) for two different scenes (**a**,**b**).

To show the effectiveness of our method more fairly, we compare our method with some state-of-the-art voxel-based methods. The detection results of Pointpillars [13], SECOND [12], and our method are visualized as shown in Figure 9.

From the point cloud detection results on the left of Figure 9a, we can see that the bounding box regression of Pointpillars [13] is not satisfactory, while SECOND [12] is more susceptible to interference from complex backgrounds, and both of them have some false detections. In comparison, our method exhibits stronger robustness and does not mistakenly detect distant background points as vehicles, thereby reducing false alarms to a certain extent. In a complex scene like Figure 9b, the vehicles are severely occluded and truncated. All three methods successfully detect vehicles in this scene. However, for the bushes and signs on the side of the highway, Pointpillars [13] has two prominently false detections, and SECOND [12] incorrectly identifies the left side guardrail as a vehicle. However, our method exhibits excellent performance and perfectly avoids background interference. The above visualization results show that our network is more robust to detection tasks in complex scenes.

## 5. Discussion

Our method is not only suitable for car detection, but also suitable for various objects in real autonomous driving scenes. Besides, our proposed one-stage detector fully extracts and integrates different levels of BEV feature information, so it can be used to generate high-quality proposals and be expanded into a higher-precision two-stage detector. Of course, our method also has some shortcomings: (1) as shown in Section 4, we only compared the 3D and BEV detection performance of cars, but not pedestrians and cyclists. This is because the detection accuracy of pedestrians and cyclists has not been significantly improved. For small targets that are easily overlooked, it is not enough to use point cloud BEV information for optimization. On the one hand, these small targets contain fewer point clouds, which are easily ignored or affected by complex backgrounds. On the other hand, unlike rigid objects such as cars, the point cloud contours of pedestrians are usually complex and changeable, which also brings some challenges to anchor-based methods. (2) As shown in Table 1, the inference speed of our proposed detector can reach 11FPS on a single GTX 1080Ti GPU. However, for vehicles traveling at high speed in real scenes, such inference speed is still insufficient to complete the detection and tracking tasks. As stated by Gaussian YOLOv3 [33], a real-time detection speed of above 30 FPS is a prerequisite for autonomous driving applications. To this end, we tried to perform inference on a better-performing GPU. Although the inference speed can reach 22 FPS on TITAN XP GPU, there is still a certain gap with industrial model deployment. For the above two problems, we will consider fusing images and point clouds to improve the detection of small targets and long-distance targets. Moreover, for the deployment of our model, we will try to use TensorRT to accelerate the inference of the model and realize the detection of high-speed vehicles.

With the development of autonomous driving technology, model deployment requires detectors to have stronger generalization ability, but we observe that most of the state of the arts are only trained and evaluated on the KITTI benchmark, which is not conducive to the application of 3D object detection technology in the industrial field. Recently, more and more datasets are open to the public, such as Waymo and nuScence. To enrich the diversity of training scenarios, we need to design a standardized and unified 3D object detection framework to cleverly combine these datasets and improve the generalization ability of the model. This is also an inevitable development trend in the field of autonomous driving.

## 6. Conclusions

SECOND [12] proposes a pioneering one-stage detection framework, which uses a 3D sparse convolutional backbone network to learn the information of the point cloud space, and then converts it into a pseudo-image and uses a simple 2D RPN network to detect the object. The method based on point cloud voxels shows a unique speed advantage. Many state-of-the-art methods carry out follow-up work based on SECOND [12]. They are mainly divided into two directions: (1) optimizing SECOND [12] and designing a novel one-stage detector. For example, Pointpillars [13] and TANet [14] simplify the encoding method of SECOND [12]. They chose to abandon the 3D sparse convolutional backbone network, and use a pillar encoder to convert point clouds into a pseudo-image of the BEV, and finally use the 2D backbone network to generate the detection results. This method has the advantage of high efficiency, but due to partial loss of point cloud information, it has a performance bottleneck. (2) Taking SECOND [12] as the baseline and expanding it to a two-stage detector. For example, PartA2 [34] and PV-RCNN [35] use SECOND [12] as the first stage of the detector and refine the high-quality proposals in the second stage. This type of method has a complicated structure and requires longer training and inference time on the same GPU.

Most of the existing 3D object detection methods belong to the second category. However, our work belongs to the first category. In this paper, we introduce a new BEV feature extraction network, which uses the PSA module to ingeniously fuse multi-scale feature maps and enhance the expression ability of BEV features. Although the BEV pseudo-image obtained by the 3D backbone network is only one-eighth the size of the real scene, the feature map is still very sparse for 3D object detection. We find that simple multi-scale feature fusion does not show its due advantages, but after full cross-layer fusion, it can give full play to its advantages of information fusion. Extensive experiments on the challenging KITTI dataset show that our method has better performance in both 3D and BEV object detection compared with some previous state-of-the-art methods.

We can find that our method shows certain limitations in detecting small objects. Therefore, our future research direction is to design a multi-sensor fusion method with a faster detection speed to improve the detection performance of small objects. We observe that limited by the scanning resolution of LiDAR, small targets and distant objects usually have very sparse point clouds. We consider fusing color image data to optimize the detector. Due to the fundamental modal differences between images and point clouds, directly fusing cross-modal data usually has obvious performance bottlenecks. This is also the primary reason why the current multi-sensor methods are not effective. To this end, we will devote ourselves to exploring more effective multi-sensor fusion methods with a unified modality, such as generating high-quality pseudo-point clouds from color images and use them for point cloud completion of distant targets. For real point cloud processing, we will also introduce point cloud attention and voxel attention to avoid sampling background points or noise points. Furthermore, for the speed defects of model deployment, we will try to use ONNX-TensorRT to accelerate the inference of the model on industrial computers.

## Figures and Tables

**Table 1:** 3D detection and bird’s eye view detection performance: Average precision (AP) (%) of the car on the KITTI validation set.

Type	Method	Modality	3D Detection (IoU = 0.7)			BEV Detection (IoU = 0.7)			FPS
			Easy	Moderate	Hard	Easy	Moderate	Hard	
	MV3D [20]	RGB + LiDAR	71.29	62.68	56.56	86.55	78.10	76.67	3
	AVOD-FPN [21]	RGB + LiDAR	84.41	74.44	68.65	N/A	N/A	N/A	10
2-stage	F-PointNet [22]	RGB + LiDAR	83.76	70.92	63.65	88.16	84.02	76.44	6
	IPOD [30]	RGB + LiDAR	84.10	76.40	75.30	88.30	86.40	84.60	N/A
	PointRCNN [25]	LiDAR	88.88	78.63	77.38	N/A	N/A	N/A	10
	VoxelNet [11]	LiDAR	81.97	65.46	62.85	89.60	84.81	78.57	4
	SECOND [12]	LiDAR	87.43	76.48	69.10	89.96	87.07	79.66	25
	Pointpillars [13]	LiDAR	86.13	77.03	72.43	89.93	86.92	84.97	62
1-stage	3DBN [31]	LiDAR	87.98	77.89	76.35	N/A	N/A	N/A	8
	TANet [14]	LiDAR	88.21	77.85	75.62	N/A	N/A	N/A	29
	Voxel-FPN [32]	LiDAR	88.27	77.86	75.84	**90.20**	**87.92**	**86.27**	50
	PSANet (Ours)	LiDAR	**89.02**	**78.70**	**77.57**	**90.20**	87.88	86.20	11

**Table 2:** Performance comparison of different backbone networks on the KITTI validation set.

Method	3D Detection (IoU = 0.7)			BEV Detection (IoU = 0.7)		
	Easy	Moderate	Hard	Easy	Moderate	Hard
Baseline	88.46	78.15	76.68	90.01	87.45	85.08
Baseline+Coarse Branch	88.73	78.24	76.50	90.07	87.51	85.56
Baseline+Fine Branch	88.52	77.98	76.62	90.15	87.63	86.14
Baseline+PFH-PSA	**89.02**	**78.70**	**77.57**	**90.20**	**87.88**	**86.20**
*Improvement*	*+0.56*	*+0.55*	*+0.89*	*+0.19*	*+0.43*	*+1.12*

**Table 3:** Performance comparison of different fusion methods on the KITTI validation set.

Method	3D Detection (IoU = 0.7)			BEV Detection (IoU = 0.7)		
	Easy	Moderate	Hard	Easy	Moderate	Hard
Baseline	88.46	78.15	76.68	90.01	87.45	85.08
Early Concat Fusion	88.44	78.27	77.09	90.10	87.83	86.28
Early Sum Fusion	**89.10**	78.68	77.35	**90.21**	87.82	85.99
Late Concat Fusion	88.66	78.55	77.25	90.12	**88.05**	**86.80**
Late Sum Fusion	89.02	**78.70**	**77.57**	90.20	87.88	86.20

## Data Availability

Publicly available datasets were analyzed in this study. This data can be found here: http://www.cvlibs.net/datasets/kitti/eval_object.php?obj_benchmark=3d.

## Abbreviations

The following abbreviations are used in this manuscript:

BEV	Bird’s Eye View
PFH	Pyramidal Feature Hierarchy
PSA	Pyramid Splitting and Aggregation
RPN	Region Proposal Network
VFE	Voxel Feature Encoding

## References

[1] Girshick R. Fast r-cnn. Proceedings of the IEEE International Conference on Computer Vision.

[2] Liu W., Anguelov D., Erhan D., Szegedy C., Reed S., Fu C.Y., Berg A.C. Ssd: Single shot multibox detector. Proceedings of the European Conference on Computer Vision.

[3] Redmon J., Divvala S., Girshick R., Farhadi A. You only look once: Unified, real-time object detection. Proceedings of the IEEE Conference on Computer Vision and Pattern Recognition.

[4] Long J., Shelhamer E., Darrell T. Fully convolutional networks for semantic segmentation. Proceedings of the IEEE Conference on Computer Vision and Pattern Recognition.

[5] Chen L.C., Papandreou G., Kokkinos I., Murphy K., Yuille A.L. (2014). Semantic image segmentation with deep convolutional nets and fully connected crfs. arXiv.

[6] Lin G., Milan A., Shen C., Reid I. Refinenet: Multi-path refinement networks for high-resolution semantic segmentation. Proceedings of the IEEE Conference on Computer Vision and Pattern Recognition.

[7] Dong C., Loy C.C., He K., Tang X. Learning a deep convolutional network for image super-resolution. Proceedings of the European Conference on Computer Vision.

[8] Kim J., Kwon Lee J., Mu Lee K. Accurate image super-resolution using very deep convolutional networks. Proceedings of the IEEE Conference on Computer Vision and Pattern Recognition.

[9] Lim B., Son S., Kim H., Nah S., Mu Lee K. Enhanced deep residual networks for single image super-resolution. Proceedings of the IEEE Conference on Computer Vision and Pattern Recognition Workshops.

[10] Janai J., Güney F., Behl A., Geiger A. (2020). Computer vision for autonomous vehicles: Problems, datasets and state of the art. Found. Trends Comput. Graph. Vis..

[11] Zhou Y., Tuzel O. Voxelnet: End-to-end learning for point cloud based 3d object detection. Proceedings of the IEEE Conference on Computer Vision and Pattern Recognition.

[12] Yan Y., Mao Y., Li B. (2018). Second: Sparsely embedded convolutional detection. Sensors.

[13] Lang A.H., Vora S., Caesar H., Zhou L., Yang J., Beijbom O. Pointpillars: Fast encoders for object detection from point clouds. Proceedings of the IEEE Conference on Computer Vision and Pattern Recognition.

[14] Liu Z., Zhao X., Huang T., Hu R., Zhou Y., Bai X. TANet: Robust 3D Object Detection from Point Clouds with Triple Attention. Proceedings of the Thirty-Fourth AAAI Conference on Artificial Intelligence.

[15] Zhang D., Zhang H., Tang J., Wang M., Hua X., Sun Q. (2020). Feature Pyramid Transformer. arXiv.

[16] Chen X., Kundu K., Zhang Z., Ma H., Fidler S., Urtasun R. Monocular 3d object detection for autonomous driving. Proceedings of the IEEE Conference on Computer Vision and Pattern Recognition.

[17] Li B., Ouyang W., Sheng L., Zeng X., Wang X. Gs3d: An efficient 3d object detection framework for autonomous driving. Proceedings of the IEEE Conference on Computer Vision and Pattern Recognition.

[18] Ma X., Wang Z., Li H., Zhang P., Ouyang W., Fan X. Accurate monocular 3d object detection via color-embedded 3d reconstruction for autonomous driving. Proceedings of the IEEE International Conference on Computer Vision.

[19] Qi C.R., Su H., Mo K., Guibas L.J. Pointnet: Deep learning on point sets for 3d classification and segmentation. Proceedings of the IEEE Conference on Computer Vision and Pattern Recognition.

[20] Chen X., Ma H., Wan J., Li B., Xia T. Multi-view 3d object detection network for autonomous driving. Proceedings of the IEEE Conference on Computer Vision and Pattern Recognition.

[21] Ku J., Mozifian M., Lee J., Harakeh A., Waslander S.L. Joint 3d proposal generation and object detection from view aggregation. Proceedings of the 2018 IEEE/RSJ International Conference on Intelligent Robots and Systems (IROS).

[22] Qi C.R., Liu W., Wu C., Su H., Guibas L.J. Frustum pointnets for 3d object detection from rgb-d data. Proceedings of the IEEE Conference on Computer Vision and Pattern Recognition.

[23] Liang M., Yang B., Wang S., Urtasun R. Deep continuous fusion for multi-sensor 3d object detection. Proceedings of the European Conference on Computer Vision (ECCV).

[24] Qi C.R., Yi L., Su H., Guibas L.J. Pointnet++: Deep hierarchical feature learning on point sets in a metric space. Proceedings of the Advances in Neural Information Processing Systems.

[25] Shi S., Wang X., Li H. Pointrcnn: 3d object proposal generation and detection from point cloud. Proceedings of the IEEE Conference on Computer Vision and Pattern Recognition.

[26] Girshick R., Donahue J., Darrell T., Malik J. Rich feature hierarchies for accurate object detection and semantic segmentation. Proceedings of the IEEE Conference on Computer Vision and Pattern Recognition.

[27] Yang B., Luo W., Urtasun R. Pixor: Real-time 3d object detection from point clouds. Proceedings of the IEEE conference on Computer Vision and Pattern Recognition.

[28] Lin T.Y., Goyal P., Girshick R., He K., Dollár P. Focal loss for dense object detection. Proceedings of the IEEE International Conference on Computer Vision.

[29] Geiger A., Lenz P., Urtasun R. Are we ready for autonomous driving? the kitti vision benchmark suite. Proceedings of the IEEE Conference on Computer Vision and Pattern Recognition.

[30] Yang Z., Sun Y., Liu S., Shen X., Jia J. (2018). Ipod: Intensive point-based object detector for point cloud. arXiv.

[31] Li X., Guivant J., Kwok N., Xu Y., Li R., Wu H. (2019). Three-dimensional Backbone Network for 3D Object Detection in Traffic Scenes. arXiv.

[32] Kuang H., Wang B., An J., Zhang M., Zhang Z. (2020). Voxel-FPN: Multi-Scale Voxel Feature Aggregation for 3D Object Detection from LIDAR Point Clouds. Sensors.

[33] Choi J., Chun D., Kim H., Lee H.J. Gaussian yolov3: An accurate and fast object detector using localization uncertainty for autonomous driving. Proceedings of the IEEE International Conference on Computer Vision.

[34] Shi S., Wang Z., Shi J., Wang X., Li H. (2020). From points to parts: 3d object detection from point cloud with part-aware and part-aggregation network. IEEE Trans. Pattern Anal. Mach. Intell..

[35] Shi S., Guo C., Jiang L., Wang Z., Shi J., Wang X., Li H. Pv-rcnn: Point-voxel feature set abstraction for 3d object detection. Proceedings of the IEEE/CVF Conference on Computer Vision and Pattern Recognition.

